# Analysis of the Microstructure and Mechanical Properties of Titanium-Based Composites Reinforced by Secondary Phases and B_4_C Particles Produced via Direct Hot Pressing

**DOI:** 10.3390/ma10111240

**Published:** 2017-10-27

**Authors:** Isabel Montealegre-Melendez, Cristina Arévalo, Enrique Ariza, Eva M. Pérez-Soriano, Cristina Rubio-Escudero, Michael Kitzmantel, Erich Neubauer

**Affiliations:** 1 Department of Engineering and Materials Science and Transportation, School of Engineering, Universidad de Sevilla, Camino de los Descubrimientos s/n, 41092 Seville, Spain; imontealegre@us.es (I.M.-M); evamps@us.es (E.M.P.-S.); 2 RHP-Technology GmbH, Forschungs-und Technologiezentrum, 2444 Seibersdorf, Austria; enrique.ariza@rhp-technology.com (E.A.); michael.kitzmantel@rhp-technology.com (M.K.); erich.neubauer@rhp-technology.com (E.N.); 3 Department of Computer Languages and Systems, Universidad de Sevilla, Avenida Reina Mercedes s/n, 41012 Seville, Spain; crubioescudero@us.es

**Keywords:** titanium matrix composites, powder metallurgy, hot pressing, mechanical properties, microstructure, in-situ reaction.

## Abstract

In the last decade, titanium metal matrix composites (TMCs) have received considerable attention thanks to their interesting properties as a consequence of the clear interface between the matrix and the reinforcing phases formed. In this work, TMCs with 30 vol % of B_4_C are consolidated by hot pressing. This technique is a powder metallurgy rapid process. Incorporation of the intermetallic to the matrix, 20 vol % (Ti-Al), is also evaluated. Here, the reinforcing phases formed by the reaction between the titanium matrix and the ceramic particles, as well as the intermetallic addition, promote substantial variations to the microstructure and to the properties of the fabricated composites. The influences of the starting materials and the consolidation temperature (900 °C and 1000 °C) are investigated. By X-ray diffraction, scanning and transmission electron microscopy analysis, the in-situ-formed phases in the matrix and the residual ceramic particles were studied. Furthermore, mechanical properties are studied through tensile and bending tests in addition to other properties, such as Young’s modulus, hardness, and densification of the composites. The results show the significant effect of temperature on the microstructure and on the mechanical properties from the same starting powder. Moreover, the Ti-Al addition causes variation in the interface between the reinforcement and the matrix, thereby affecting the behaviour of the TMCs produced at the same temperature.

## 1. Introduction

In-situ reinforced titanium matrix composites (TMCs) are considered interesting materials thanks to excellent properties such as high strength and high stiffness [1,2]. Previous research has demonstrated the advantage of these composites, in which the interface between the reinforcement and matrix is clear, enhancing the specific strength and high-temperature durability [3,4,5,6,7,8,9,10]. Among different candidates to reinforce the titanium matrix, TiC, TiB and TiB_2_ are proposed as the most suitable due to their thermodynamic stability and similar coefficient of thermal expansion (CTE) [11]. Various authors have described particles of B_4_C and the titanium matrix as a suitable combination for producing in-situ reinforced composites. The B_4_C particles are the source of secondary phases and compounds responsible for the strengthening of the materials [12,13,14].

Furthermore, previous authors reveal the advantage of adding aluminium when forming intermetallic compounds [15]. The Ti-Al system is considered an interesting combination of materials in aerospace sector. The employ of Ti-Al composites provides high tensile strength and an acceptable elongation (1–2%) [16].The properties that these intermetallic compounds exhibit are also particularly attractive for lightweight and high-temperature structural applications. In terms of TMCs, the intermetallic compounds have been considered as to enhancing specific stiffness and hardness [3,17,18,19]. Therefore, the incorporation in the titanium matrix of intermetallics and particles of B_4_C modifies, to a large extent, the microstructure and the final properties [13,15,20].

From the manufacturing process point of view, these in-situ composites have been fabricated by diverse routes in recent years [21,22,23,24]. Powder metallurgy techniques are considered to be interesting fabrication methods to produce in-situ TMCs [1,25,26,27,28]. At the present time, powder metallurgy enables researchers to advance further in this field thanks to its versatility and flexibility. Techniques, such as spark plasma sintering and hot pressing, are two possible candidates to produce in-situ TMCs, due to their short run cycle and the high heating rate reached during the process [28,29]. All previous studies show that temperature is one of the most important factors in promoting the formation of the secondary phases in the matrix. As expected, the reactions between the matrix and the B and C sources depend upon the temperature, in addition to the processing time. This has been widely reported in recent research work [15]. However, there are insufficient studies to clarify the influence of the starting materials, such as the incorporation of intermetallics into the matrix. Therefore, the present study focuses on the relationship between the final properties and substantial changes in the TMC microstructures deriving from the starting materials and the processing temperature. In this context, the development and manufacturing of materials with highly specific properties for aerospace applications render their study and investigation attractive.

## 2. Experimental

### 2.1. Starting Materials

**Figure 1:**
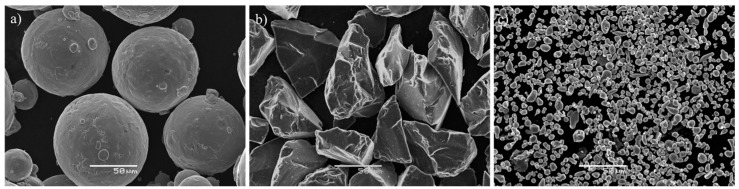
SEM (scanning electron microscopy) images of the raw materials: (**a**) Ti Grade 1 powders; (**b**) B_4_C powders; and (**c**) Ti-Al powders.

Commercial titanium grade 1 was used as matrix powder. This titanium powder showed spherical morphology with a size range of 75–180 µm, and it was manufactured by AP&C (Quebec, QC, Canada). B_4_C particles, with irregular and angular shapes and an average particle size of 45–75 µm, were used as source of B and C for in-situ-formed TiB and TiC (purity ≥ 98%); it was supplied by ABCR GmbH & Co., KG (Karlsruhe, Germany). The in-situ-formed Ti_x_Al_y_ intermetallic was made from elemental blending, as raw materials: a fine titanium powder with a size range of 20–45 µm and aluminium powder with a purity of 99.7% and an average size of 6 µm, both with an irregular round shape and manufactured by TLS GmbH (Bitterfeld, Germany) and NMD GmbH (Heemsen, Germany), respectively. From a point of view of the reactivity between titanium and aluminium, titanium fine powder was selected because small particle-size powder involves a high diffusion rate, which is beneficial to Ti-Al reaction. Scanning electron microscopy (SEM) images of the powders were taken by a JEOL 4640LV (Tokyo, Japan), equipped with energy dispersive spectroscopy (EDS) to verify the morphologies of the starting powder materials previously described. Figure 1 presented SEM images of the raw materials.

### 2.2. Direct Hot Pressing

Before the direct hot pressing process (dHP), the powder mixtures were prepared. Firstly, the weight percentage of the Ti-Al blend was made of 64% fine titanium particles and 36% aluminium powder. It was developed according to the atomic ratio Ti:Al. The mixing stage was carried out in a Sintris mixer (Sintris Macchine S.R.L., Piacenza, Italy) for 12 h, using ZrO_2_ balls (Ø 3 mm) in cyclohexane, with a speed of 60 rpm. The weight ratio of ceramic balls and powder was 10:1. Next, the drying of the Ti-Al powder took place in a vacuum oven at 100 °C for six hours to evaporate the solvent; after that step, powders were blended for two hours in the same mixer. Once the Ti-Al powder blending was complete, the mix of the starting materials of the in-situ TMCs was carried out. The content of B_4_C particles was 30 vol % in all the specimens. Twenty vol % of intermetallic was used in one of the two types of TMCs. A dry mixing stage of the starting powders was carried out for two hours in the Sintris mixer at a speed of 60 rpm. They were subsequently hot consolidated into plates with a diameter of 80 mm and a height of 4 mm by a fast consolidation system. For this direct hot pressing process, a special hot pressing machine was used (dHP equipment of RHP-Technology GmbH and Co., KG, Seibersdorf, Austria). This equipment provided a major advantage thanks to its high heating rate, due to its direct heating of the die, up to 400 K·min^−1^. The die employed for all of the dHP experiments was made of graphite (punch Ø 80 mm). For each consolidation test, the die was striped with thin graphite paper with a protective coating of boron nitride (BN). The fixed processing parameters were: heating rate 100 °C∙min^−1^ and a 60 Hz, AC current. The mechanical pressure was 35 MPa in vacuum conditions (10^−1^ mbar). In order to evaluate the temperature influence on the behaviour of the in-situ TMCs, two temperature values were tested for the powder mix without intermetallic: 900 °C and 1000 °C, with holding time 15 min. The powder mix with 20 vol % of Ti-Al was hot consolidated at 1000 °C for 15 min, at 35 MPa in the same vacuum conditions (10^−1^ mbar) (see Table 1). After the dHP process, samples were removed from the die and cleaned by sand blasting to completely remove the graphite paper residues from the surface. The specimens were then prepared to be characterized.

### 2.3. Specimen Characterization

Phase analysis was conducted after careful polishing of the sample surface. The phase compositions of the specimens were identified by X-ray diffraction (XRD-Bruker D8 Advance A25, Billerica, MA, USA) using Cu-K_α_ (λ = 1541 Å) radiation over the angular range of 20°–90°. The microstructural analysis consisted of the study of the microstructure of the specimens by a scanning electron microscope (SEM JEOL 6460LV equipped with EDS, Tokyo, Japan) and a transmission electron microscope (S/TEM Talos™ F200S, Thermo Fisher Scientific, Hillsboro, OR, USA).

Mechanical properties of the composites were determined through tensile and bending tests. The specimen plates were machined into tensile test samples with standard dimensions by the ISO 6892-1:2016. Room-temperature tensile tests were conducted on a universal testing machine Instron 5505 (Norwood, MA, USA) with a strain rate of 1 mm·min^−1^. In the same testing machine, high-temperature tests at 250 °C with a cross-head speed of 1 mm·min^−1^ were also performed. Three tensile samples for the room-temperature test and two samples for the high-temperature test were machined for each consolidated material in order to obtain average values of the mechanical properties and to evaluate the reproducibility of the results.

The flexural sample dimensions were 4 mm × 3 mm × 20 mm according to EN ISO 3325:1999/A1:2002. Three specimens were also machined to calculate an average value of the flexural properties of the in-situ TMCs. These tests were performed on the same universal testing machine, Instron 5505, with a speed of 5 mm·min^−1^ at room temperature.

Archimedes’ method (ASTM C373-14) was used to determine density. Vickers hardness (HV10) of the specimens was measured by the use of a tester model Struers-Duramin A300 (Ballerup, Germany). The error of the equipment is 0.05. Eight indentations were made in each composite in order to obtain the average composite hardness. An ultrasonic method (Olympus 38 DL, Tokyo, Japan) was employed to measure Young’s Modulus. It was used with a pulse generator/receiver, recording the transit time (outward/return) through the thickness of the sample. This technique allowed the estimation of both longitudinal (VL) and transverse (VT) propagation velocities of acoustic waves. To measure the propagation velocity correctly, the surface of the samples needed to be properly ground and polished (samples with smooth and parallel surfaces) and the delay times of transducers minimized by following an iterative measurement protocol. Young’s Modulus was calculated from the density (g·cm^−3^), VL and VT [30].

## 3. Results and Discussion

### 3.1. X-Ray Diffraction

**Figure 2:**
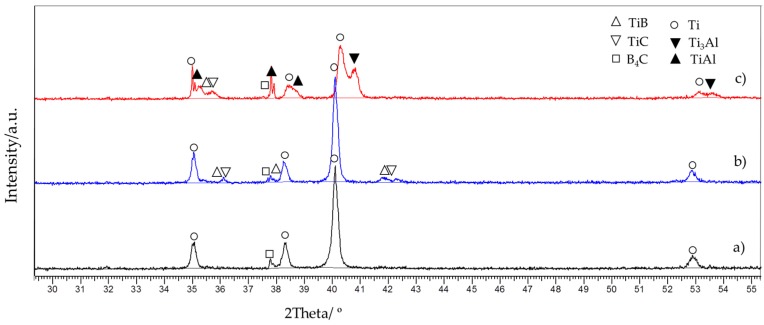
XRD (X-ray diffraction) patterns of the in-situ TMCs (titanium matrix composites) with 30 vol % of B_4_C produced at: (**a**) 900 °C; (**b**) 1000 °C; and; (**c**) 1000 °C with 20 vol % Ti-Al.

The evolution of the in-situ-formed phases with processing temperature in the composites was followed through X-ray diffraction analysis. The XRD patterns of TMCs produced at different temperatures (900 °C and 1000 °C) and compositions (with and without 20 vol % Ti-Al) are represented in Figure 2. Diffraction peaks for B_4_C were observed in every XRD spectra pattern for all the TMCs formed in situ. This indicated that the reaction between titanium matrix and the particles was not entirely completed. However, as the temperature approached 1000 °C, the peaks became less pronounced.

The patterns of the TMCs without intermetallic showed sharp α-Ti peaks, as shown in Figure 2a,b, while TMCs with 20 vol % of Ti-Al pattern, Figure 2c, showed weak Ti peaks and revealed additional intermetallic peaks, TiAl and Ti_3_Al. This occurred in specimens consolidated at 1000 °C. This temperature was considered suitable for the evaluation of the solubility of Al in Ti, in order to study, on one hand, the phase transformation and, on the other, the solid solubility of Al in Ti [3,31,32,33]. According to the Ti-Al phase diagram, given the amount of aluminium (7 wt. %) in the specimen, the appearance of intermetallic is expected and the remaining aluminium does not precipitate but remains dissolved in the titanium matrix [34]. A shift to a higher angle in the Ti main peak in the diffraction pattern indicated that Al diffused in Ti matrix via solid state diffusion (Figure 2c). Based on these patterns, SEM images and EDS mappings confirmed that Al was a solid solution in Ti at this temperature. This phenomenon was studied in detail in previous research work [3].

TMCs produced at 900 °C did not reveal any TiC and TiB peaks as shown in Figure 2a. However, TiC and TiB diffraction peaks appeared in the diffraction patterns of TMCs consolidated at 1000 °C as shown in Figure 2b, while the XRD patterns of TiB_2_ peaks were not detected since this phase transformation required longer [20,35]. This result aligns with previous work [36]. The intensities of the TiC and TiB diffraction peaks increased when the starting powder composition of the TMCs did not contain 20 vol % of Ti-Al, which indicated that the volume fraction of in-situ-formed TiC and TiB were affected by the addition of Ti-Al. It could be seen that when there was Ti-Al in the starting materials, the TiC and TiB peaks were weak; it seems that the formation of intermetallic phases retarded the origin of secondary phases (TiB and TiC). This was verified by semi-quantitative analysis made using Reference Intensity Ratio (RIR). These results showed that in TMCs produced at 1000 °C: (i) in the specimen with intermetallic, the composition values of TiB and TiC were 2.1 wt. % and 0.3 wt. %, respectively; (ii) in the specimen without intermetallic, these figures were 2.7 wt. % and 1.1 wt. %, respectively. These results are going to be verified by SEM, EDS and TEM microstructural characterization.

### 3.2. Microstructural Analysis

A microstructural characterization was performed to evaluate, on the one hand, the influence of the processing temperature (900 °C and 1000 °C) in the microstructure of the specimens from the same starting powder (Ti + 30 vol % B_4_C) and, on the other hand, the incorporation of Ti-Al in the in-situ TMCs process at 1000 °C.

**Figure 3:**
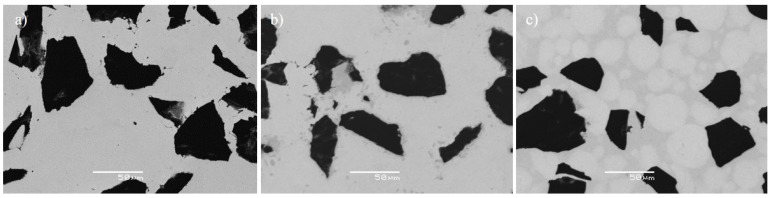
SEM images of TMCs manufactured at: (**a**) 900 °C; (**b**) 1000 °C; and (**c**) 1000°C with Ti-Al.

Figure 3 shows a comparison of the three different microstructures. In Figure 3a, no visible reaction between the matrix and the B_4_C particles at 900 °C was observed. However, the increase of the processing temperature up to 1000 °C led to the formation of secondary phases observed as darker areas around B_4_C particles. The products of these reactions could be appreciated in Figure 3b. Furthermore, in Figure 3a and b, the grain boundaries could not be easily recognized. In contrast, in Figure 3c, the limits among the grains of the matrix could be appreciated. The presence of the intermetallic in the grain boundaries considerably delimited such borders. Additionally, few pores were observed bordering the B_4_C particles in these specimens. It was not easy to differentiate the formation of secondary phases by visual analysis in Figure 3c. This phenomenon was similar to that described and studied in previous research work [3]. The addition of intermetallic slowed the formation of TiC and TiB. This was consistent with the results described previously in XRD patterns (see Figure 2).

**Figure 4:**
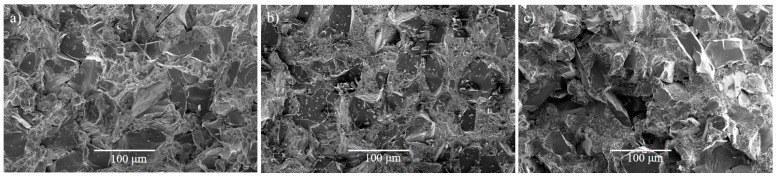
SEM images of fracture surface of composites tested at room temperature and manufactured at: (**a**) 900 °C; (**b**) 1000 °C; and (**c**) 1000 °C with Ti-Al.

Figure 4 shows the fracture surface of composites after the tensile test performed at room temperature. Dispersed phases with several sizes and shapes were found in different zones of the fracture surface. As expected, the processing time (15 min) and the low consolidation temperatures (900 °C and 1000 °C) were insufficient to achieve a complete reaction between the matrix and the B_4_C particles. Therefore, these particles were still present in the matrix, and remain easily recognizable by their dark grey colour and their angular morphology. Less brittle fracture was observed on the surface of the specimen consolidated at 900 °C (Figure 4a). However, composites consolidated at 1000 °C showed more brittle fractures. These results were in agreement with the mechanical properties (see the tensile and bending properties section).

Regarding the secondary phases formed as products of the reaction between the matrix and the B_4_C particles, a detailed study of their morphology and shapes was carried out only in the specimens manufactured at 1000 °C without intermetallic, since in these specimens, more TiC and TiB were detected by XRD (see Figure 2) and observed in SEM images. The TiC and TiB phases were evaluated at micro/nano-scale via TEM images. Moreover, this specimen was selected (from the three) because of its properties. These properties will be discussed further later.

From a micro-scale point of view, the morphology of the in-situ formed TiB and TiC showed similar traits to the characteristics described in previous research works [15,21].

**Figure 5:**
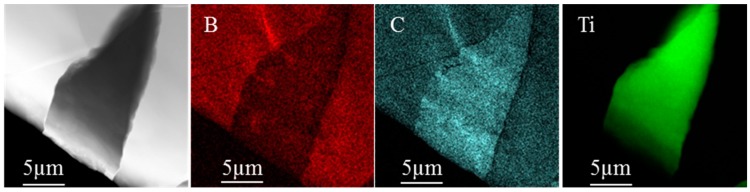
TEM image and compositional mapping of TiC formation in TMCs produced at 1000 °C for 15 min.

**Figure 6:**
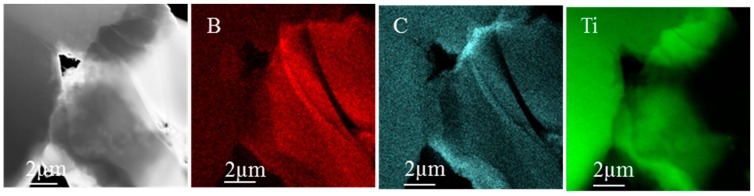
TEM image and compositional mapping of TiB and TiC formations of TMCs produced at 1000 °C for 15 min.

TEM study (Figure 5 and Figure 6) showed that in-situ TiB and TiC were formed by the reaction between the Ti matrix and B_4_C particles. The in-situ reinforcements were randomly distributed close to the boundaries of B_4_C particles. In Figure 5, it was appreciated that the TiC phase originated amongst B_4_C particles. An example of the formation of the TiB phase can be observed in Figure 6. In this TEM image, micro TiB and TiC precipitates resulted as products of the Ti and B_4_C particles interaction.

**Figure 7:**
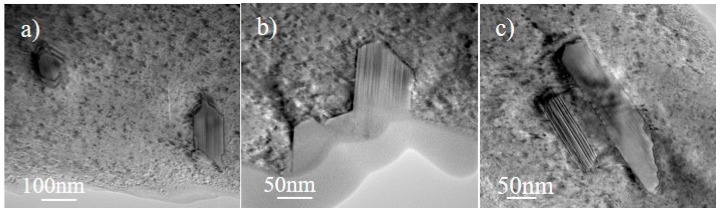
Nano-scale TEM images of precipitates: (**a**) striped polygonal shape; (**b**) striped and non-striped polygonal shape; (**c**) striped and non-striped whisker.

**Figure 8:**
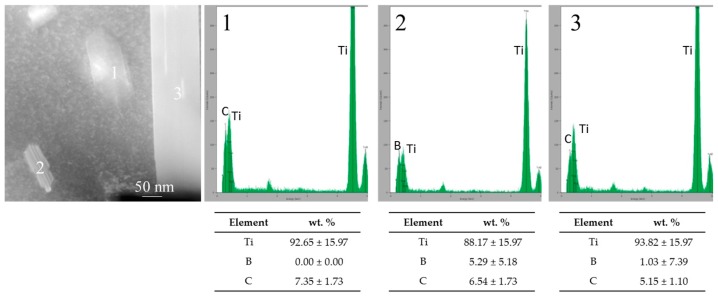
TEM images and EDS (energy dispersive spectroscopy) spectra for different precipitates in Ti matrix.

Considering the nano-scale study of the in-situ TiC and TiB, it is worth noting that B and C diffusion into the titanium grains took place. The result of this diffusion was detected as nano-precipitates in these grains. It was analysed by TEM and EDS (Figure 7 and Figure 8). Interesting morphologies and shapes were revealed for these precipitates with classification as whisker and polygonal geometries [13], which differ from striped or non-striped surfaces. Thus, four possible precipitate morphologies could be identified: (i) non-striped whisker [23], (ii) non-striped polygonal shape, (iii) striped whisker and (iv) striped polygonal shape.

Regarding the size of precipitates, similar widths were measured in polygonal shapes (40–70 nm approx.). However, the length of whiskers covered a wide range of sizes varying from 100 to 500 nm (see Figure 7 and Figure 8, spot 3).

EDS analysis revealed the compositions of these precipitates; the striped shapes corresponding to the TiB phase and the non-striped shapes corresponding to the TiC phase (see Figure 8). As such, TiB precipitates appeared as whiskers and polygonal shapes, but, in all these morphologies, a striped texture was observed. In the case of TiC, not only was polygonal form observed, but also needle-shape without striped texture (see Figure 7c). These results were in accordance with previous research works [13,23].

### 3.3. Densification, Hardness and Young’s Modulus

From a process parameters point of view, by the use of the same starting powder, the densification, and average hardness of the eight indentations made for each specimen with their corresponding standard deviation, were studied in addition to the Young’ modulus (see Table 2). This table shows that the lower the temperature, the lower the densification.

A similar tendency was observed for average composite hardness values. This could be related to the apparition of the TiC and TiB, originated through the B_4_C decomposition. Thus, an increase of the temperature to 1000 °C contributed towards enhancing density and hardness by 3% and 42%, respectively. In the same way, Young’s modulus depended on the consolidation temperature; the higher the temperature, the higher the Young’s Modulus. Regarding the hardening effect, the apparition of precipitates in the titanium grains led to the enhancement of the hardness. Under similar fabrication conditions, the influence of the starting materials was studied. In general, the composites showed higher properties than the pure titanium matrix, as was expected. Comparing the specimens produced at 900 °C, the addition of B_4_C incremented the hardness by 33.13%. At 1000 °C, the specimens made from 30 vol % of B_4_C and 20 vol % Ti-Al presented higher hardness values compared to those without Ti-Al. As mentioned in the section on microstructural study, the location of the reinforcing phases varied slightly and the formation of a secondary phase took place at 1000 °C. There were intermetallic compounds formed at the titanium grain boundaries. In this way, the hardening effect was observed in an enhancement of 16.3%. The Ti-Al addition started a slight diminution in the densification of the specimens (1.1%). However, Young’s Modulus was not considerably affected by the incorporation of the Ti-Al and the formation of intermetallic at grain boundaries (Table 2). In agreement with previous authors [37,38], determinate strengthening mechanisms can contribute as: solution strengthening of Al, C or B in the matrix; Orowan strengthening by disperse secondary phases as TiB and TiC; and load-transferred strengthening from titanium matrix to reinforcement particles.

### 3.4. Tensile and Bending Properties

From the perspective of the strengthening behaviour, the tensile and bending properties were mainly affected by the in-situ-formed TiC and TiB, in addition to the B_4_C particles which had not reacted with the titanium matrix [13,22,39,40]. The strength and ductility of these composites were generally influenced by the processing temperature and compositions, as expected. Regarding the operational temperature, composites processed at 900 °C had higher ductile properties and lower strength than the rest of the specimens tested at room temperature. The increase of the temperature up to 1000 °C involved an increase in the stiffness of the specimens, at the expense of ductility. The ductility decrement was 45%. The strengthening effect was closely related to volume fraction, and the size and distribution of reinforcements. The effect of in-situ generated reinforcements was verified after the mechanical properties tests. According to the starting materials used, the pure matrix presented higher values of elongation than the composites processed at 900 °C and 15 min. It is closely related to the location of the B_4_C particles, as was observed in the microstructural study. The homogeneous distribution of the reinforcement particles led to blocking the motion of the dislocation through the titanium matrix thereby promoting the strengthening effect at the expense of its ductility behaviour. Regarding the specimens consolidated at 1000 °C, there were differences in the tensile properties due to the incorporation of the Ti-Al in the starting materials. There was low deformation measured in specimens in which intermetallic compounds were observed, and was caused by the presence of intermetallic phases in the grain boundaries. This compound acted as a barrier and contributed to the embrittlement of the specimens.

On evaluating the tensile properties measured at 250 °C, the same trend is observed. In general, the values of the deformation of composites were higher than those measured at room temperature that included the decrement of the ultimate tensile strength. However, there was 18% of ductility loss in specimens produced at 1000 °C with respect to those at 900 °C, when they were tested at 250 °C. This means that there is little difference between the ductility behaviours of the specimens. Three point flexural test results are presented in Table 3. The ductility values showed that the specimen produced at 900 °C was the most ductile compared to the others. The increase of the temperature caused an embrittlement effect. The composite whose starting powder had no Ti-Al possessed better properties than the others produced under similar operational conditions.

## 4. Conclusions

TMCs were processed by direct hot pressing for 15 min at 900 °C and 1000 °C. The temperature effect was evaluated in the system Ti-B_4_C in order to identify the in-situ-formed TiC-TiB and the residual B_4_C. Moreover, at 1000 °C, the effect of the intermetallic in the TMCs was investigated. Several conclusions can be drawn from the study:

- Composites manufactured at 900 °C did not present secondary phases; therefore, the strengthening effect of the in-situ-formed TiC-TiB remained undetected. Meanwhile the TMCs manufactured at 1000 °C showed improvement of the tensile strength at the expense of a reduction in ductile properties.
- Considering the starting powder as an influencing factor, the origination of intermetallics slowed the in-situ TiC and TiB formed under the same processing conditions (1000 °C). This was confirmed by semi-quantitative analysis (RIR). Thus, TMCs from starting powder with Ti-Al presented better hardness and Young’s modulus; however, they possessed lower mechanical behaviour than composites without intermetallics.
- Investigation of the in-situ-formed TiC and TiB revealed different shapes, morphologies and sizes in addition to the distribution of these reinforcements. Dispersion and distribution of such precipitates may affect the final properties of the specimens.

The presented results suggest that direct hot pressing might be suitable for the development of in-situ TMCs that employ temperatures from 1000 °C. No completed reaction between the B_4_C particles and the matrix took place.

## Figures and Tables

**Table 1:** In-situ TMCs (titanium matrix composites) processed at different manufacturing conditions.

TMCs Composition	Temperature (°C)	Time (min)	Pressure (MPa)
Ti + 30 vol % B_4_C	900	15	35
Ti + 30 vol % B_4_C	1000	15	35
Ti+30 vol % B_4_C + 20 vol % Ti-Al	1000	15	35

**Table 2:** Density, hardness and Young´s modulus of the composites.

Materials	Theo. Density (g·cm^−3^)	Arch. Density (g·cm^−3^)	Densification (%)	Average Composite Hardness (HV10)	Young´s Modulus (GPa)
Ti matrix-900 °C	4.51	4.47	99.11	181.10 ± 5.71	85.67 ± 29.13
Ti + 30 vol % B_4_C-900 °C	3.91	3.77	96.48	241.10 ± 6.65	134.21 ± 5.62
Ti + 30 vol %B_4_C-1000 °C	3.91	3.87	99.07	342.40 ± 20.23	165.84 ± 4.64
Ti + 30 vol % B_4_C + 20 vol % Ti-Al-1000 °C	3.79	3.71	97.98	398.20 ± 23.41	164.42 ± 0.93

**Table 3:** Tensile properties measured at room temperature and 250 °C and bending properties.

Materials	Tensile Properties				Bending Properties	
	Room Temperature		250 °C		Room Temperature	
	σ_UTS_ (MPa)	ε_max_ (%)	σ_UTS_ (MPa)	ε_max_ (%)	σ_UBS_ (MPa)	ε_max_ (%)
cp Ti-900 °C	301.23	20.45	-	-	-	-
Ti + 30 vol % B_4_C-900 °C	323.42	0.42	248.48	12.31	636.78	1.501
Ti + 30 vol % B_4_C-1000 °C	425.84	0.23	311.04	10.02	511.30	1.091
Ti + 30 vol % B_4_C + 20 vol % Ti-Al-1000 °C	331.70	0.21	210.21	5.55	567.19	1.471
